# Microalgae Parasite Diseases of *Mytilus galloprovincialis*: Infections, Immunology and Antioxidant Defense

**DOI:** 10.3390/antiox14121430

**Published:** 2025-11-28

**Authors:** Daria Lavrichenko, Elina Chelebieva, Elizaveta Bogacheva, Ekaterina Vodiasova, Victoria Uppe, Ekaterina Kladchenko

**Affiliations:** 1 A.O. Kovalevsky Institute of Biology of the Southern Seas of RAS, 299011 Sevastopol, Russia; 2 Federal State Funded Institution of Science «The Labor Red Banner Order Nikita Botanical Gardens-National Scientific Center of the RAS», 109992 Moscow, Russia

**Keywords:** parasitic microalgae, *Mytilus galloprovincialis*, immune response, antioxidant defense, lipid peroxidation, DNA damage, bivalve aquaculture, Black Sea

## Abstract

*Coccomyxa parasitica*-like algae pose a growing threat to bivalve aquaculture. In this work, for the first time under controlled conditions, the effect of the green parasitic microalgae of genus *Coccomyxa* sp. in the Sea of Japan on the immune and antioxidant protection of *Mytilus galloprovincialis* was studied in two ways of infection—through filtration (with feed) and injection (into an adductor). By day 7, mortality in both experimental groups reached 68%. The phagocytic activity of hemocytes significantly decreased in the feed group, which may be due to the masking of the parasite as a food particle. Despite transcriptional activation of catalase and superoxide dismutase genes in hemocytes upon injection, a decrease in enzyme activity and an increase in lipid peroxidation were observed in the gills, indicating local oxidative stress. Catalase activity in the gills was increased when mussels receive cells as food. DNA damage in hemocytes did not reach statistical significance. After injection, there was a significant decrease in the galectin gene expression. The data obtained confirm that *Coccomyxa* sp. is an active parasite capable of infecting the Mediterranean mussel and modulating the host’s defense systems.

## 1. Introduction

The cultivation of marine bivalves is a promising and rapidly developing industry in aquaculture [1,2]. Mussels are one of the most actively cultivated mollusks, accounting for 10.2% of global production [1]. The mussel *Mytilus galloprovincialis* Lamarck, 1819 is a highly invasive species found in many coastal areas of the World Ocean [3]. This successful adaptation is explained by the physiological characteristics of this species, which make it possible to survive in adverse conditions. However, the productivity and sustainability of mussel aquaculture are constantly under threat due to a wide range of pathogens and parasites.

At the same time, the intensification and global expansion of bivalve aquaculture is contributing to the unintended spread of invasive species and their parasites, which is exacerbated by climate change and disruption of natural barriers between ecosystems [4,5,6]. Bivalves serve as hosts for a variety of parasitic and symbiotic organisms, including gregarines of the genus *Nematopsis* (affecting the digestive gland), turbellaria *Urastoma cyprinae* (localized in the gills), haplosproid-like plasmodia, ciliates of the *Ancistrocoma*, oarfish *Mytilicola orientalis*, and endosymbiotic crabs *Pinnotheres pisum* [7,8,9,10]. These parasites and symbionts can have a significant impact on the physiological state, growth, survival, and commercial quality of mussels, threatening both the economic sustainability of aquaculture farms and the ecological safety of coastal ecosystems. A key component of the bivalve immune response to such pathogens is the production of reactive oxygen species (ROS) by hemocytes during the respiratory burst induced by phagocytosis [11]. However, excessive ROS production can cause oxidative stress, impairing host cell function and leading to apoptosis and necrosis [12]. This imbalance leads to oxidative damage to essential cellular macromolecules: lipid peroxidation disrupts cell membrane integrity, protein carbonylation impairs enzymatic function, and DNA damage can lead to cell death. Some parasites, such as *Percinsus marinus*, protect themselves from the immune defenses of the host by reducing ROS production during phagocytosis through the action of superoxide dismutase [13]. To maintain redox homeostasis, bivalves rely on a complex antioxidant system. The first line of defense involves superoxide dismutase (SOD), which catalyzes the dismutation of superoxide anion (O_2_^−^) into hydrogen peroxide (H_2_O_2_) and oxygen. Subsequently, catalase (CAT) and other peroxidases detoxify H_2_O_2_ into water and molecular oxygen, thereby preventing the formation of the highly destructive hydroxyl radical (•OH) [14]. Quantitative assessment of the activity of these enzymes, along with specific markers of oxidative damage such as lipid peroxidation products, provides a sensitive assessment of the physiological health of mollusks under pathogenic influence.

Recently, parasitic microalgae *Coccomyxa* sp. invasion has been a particular danger to mariculture farms. Frequent cases of mollusk infection by representatives of the genus *Coccomyxa* have been reported in various regions [15,16,17,18,19]. The main sign of mussel infestation is the appearance of green spots on the surface and inside the soft tissues [17]. In the study by Zuykov et al. [20], an L-shaped shell deformation is also identified as a sign, noting that such deformation is observed only in mollusks infected with *Coccomyxa*. In addition to deterioration of presentation, such disorders cause inhibition of filtration capacity, metabolic activity, the reproductive cycle, and calcification processes, having a significant impact on the growth, vitality, and survival of mollusks [18,21,22]. *Coccomyxa* sp., having a high adaptive potential, significantly expanded its range after being discovered in the Gulf of St. Lawrence, Canada [23]. The high physiological plasticity of the species, in particular, a pronounced tolerance to low salinity [24] and the ability to grow intensively in low-light conditions and a wide pH range [15], indicates a significant invasive potential, including in new ecosystems. Given the growing threat of parasitic *Coccomyxa* sp., the invasion of which causes serious damage to bivalve health, a detailed understanding of its specific pathophysiological effects is extremely necessary. In this regard, the purpose of this work was to study the effect of green parasitic algae of the genus *Coccomyxa* sp. in the Sea of Japan on the functional parameters and key health indicators of *M. galloprovincialis*. For this, we studied the interactions between *Coccomyxa* cells and Mediterranean mussels under strictly controlled experimental conditions. The inoculation was carried out in two ways: (1) orally, by introducing the algae as a feed, and (2) by microinjection of the cell suspension directly into the adductor. We evaluated the immune response in hemocytes using flow cytometry (metabolic activity and reactive oxygen species production) and DNA comet assay (DNA damage), the antioxidant enzyme activity (CAT and SOD) and lipid peroxidation (TBARS) in tissues, and quantitative assessment of the expression level of key immune system genes (lectin and galectin) and antioxidant complexes (SOD and CAT) using real-time PCR. This comprehensive assessment is crucial for understanding the pathogenicity mechanisms of this parasitic microalga and for assessing the potential risks it poses to Black Sea mussel aquaculture.

## 2. Materials and Methods

### 2.1. Experimental Animals and Conditioning

Mediterranean mussels *M. galloprovincialis* with a shell length 11.4 ± 0.6 cm and a total weight of 23.6 ± 2.4 g were collected at a local shellfish farm (n = 240). The mussels were acclimatized to laboratory conditions for 7 days (water temperature 18.0 ± 0.5 °C, salinity 18.5 ± 1.3‰, oxygen content 7.9–8.4 mg/L) in a plastic aquarium (40 mussels per 10 L) with water flow and aeration.

### 2.2. Algae Strains, Growth Conditions, and Suspension Preparation

Parasitic microalgae *Coccomyxa* sp. culture was isolated from an infected bivalve *Modiolus kurilensis* (Mytilidae). *M. kurilensis* were collected in the Peter the Great Bay of the Sea of Japan at a depth of 1.5–2 m (42°53′17.0″, 132°43′17.8″) [17,18]. Infected mollusks were characterized by pronounced green pigmentation of soft tissues. To obtain a culture of parasitic microalgae, an effective method of isolating algal cells from mollusks was used, using mechanical and enzymatic disaggregation of tissues followed by fractionation in a Percoll density gradient [19]. Briefly, axenic cultures were obtained by streaking onto an agarized (1.5–2%) F/2 medium, followed by transfer of individual colonies to a liquid F/2 nutrient medium [25]. To prevent microbial contamination, bacterial growth was controlled using a synergistic antibiotic mixture of ampicillin (Belmedpreparaty, Russia; 700 µg/mL) and cefotaxime (Biochemist, Russia; 200 µg/mL), selected for its broad-spectrum efficacy against common laboratory contaminants [26]. The laboratory microalgae cultivation was performed under controlled conditions using Feron LB-213 18 W, 175–265 V, 6400 K (Feron, Russia), providing an irradiation intensity of 85 µmol photons m^−2^ s^−2^ in a 12 h light/dark photoperiod. The growth of algae cultures was recorded by the number of cells using a MACSQuant Analyzer 10 flow cytometer (Miltenyi Biotec, Germany). Microalgae cells were acclimatized to the salinity of the Black Sea for 6 weeks, reducing the salinity of the medium from 35 to 18.

### 2.3. Mussel Infection and Hemolymph Sampling (Experimental Design)

**Figure 1:**
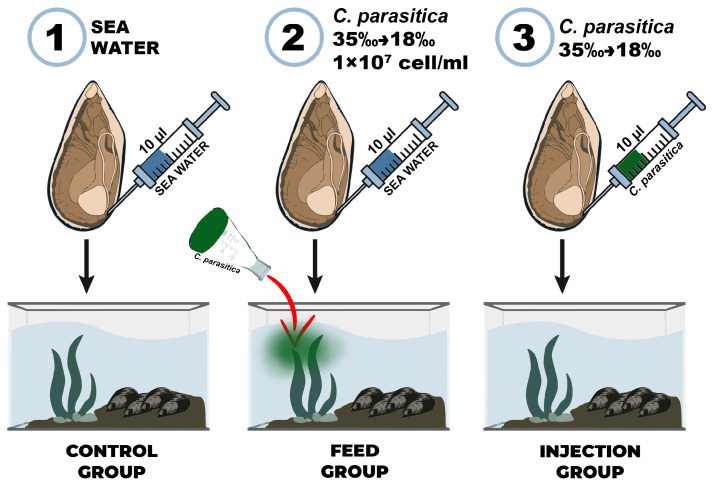
Experimental scheme of *Mytilus galloprovincialis* infection with the microalga *Coccomyxa* sp.: (1) control group, (2) feed group, and (3) injection group.

240 mussels were used in the experiment, divided into 3 groups: control (n = 80), feed (n = 80), and injection (n = 80). The control group was injected with 10 µL of sterile seawater into the adductor, and the experimental group was injected with 10 µL of *Coccomyxa* sp. adapted to the Black Sea salinity (18‰). A microalgae mixture with a concentration of 1 × 10^7^ cells/mL was added to the mussels from the feed group (Figure 1). The presence of microalgae cells in *M. galloprovincialis* hemocytes was determined using an Olympus CX43 light microscope with an Olympus DP32 camera (Olympus, Japan). Hemolymph samples were taken from individuals of all groups on the 7th day of the experiment from the adductor using a sterile syringe. The cell suspension was washed three times with sterile seawater by centrifugation (500× *g*, 5 min). To measure the activity of antioxidant enzymes, *M. galloprovincialis* gills were frozen immediately after sampling.

### 2.4. Genotoxicity

The assessment of DNA damage in hemolymph cells was performed using the DNA comet assay, according to the protocol described by Møller et al. [27]. Hemocyte suspensions were mixed with agarose and layered on an ice-cooled microscope slide (ApexLab, Russia). The glasses were incubated for 1 h at 4 °C in the dark in a lysing buffer (10 µM Tris-HCl, 100 µM EDTA, 2.5 M NaCl, 10% DMSO, 10% Triton X-100; pH 10). After incubation, the glass was subjected to gel electrophoresis at 13 V (300 mA) for 20 min in a buffer (300 M NaOH, 1 µM EDTA), with final neutralization for 15 min with neutralized buffer (0.4 M Tris-HCl; pH 7.4). The cells were stained with propidium iodide (PI). The resulting “comets” were photographed using a fluorescence microscope with magnification ×400 (Olympus CX43, Germany). The analysis was performed using the CometScore v.1.5 program (TriTek Corp., USA) by calculating the percentage of DNA in the tail, tail length and tail moment.

### 2.5. Hemocyte Functional Status

Flow cytometry analysis was performed with MACSQuant (Miltenyi Biotec, Germany). Cell debris and bacteria were eliminated on the basis of the FSC threshold, and 10,000 events were counted for each sample. Data was analysed with the MACS Quantify Software 2.13 (Miltenyi Biotec, Germany).

Reactive oxygen species (ROS) production was measured in hemocytes using the fluorescent probe 2′,7′-dichlorodihydrofluorescein diacetate (DCFH-DA), a fluorescent probe that can be used to detect intracellular levels of ROS production [19]. Cell-permeable non-fluorescent DCF is transformed by cytoplasmic esterases and then DCF reacts with intracellular ROS, forming highly fluorescent 2′,7′-dichlorofluorescein (ex: 485 nm и em: 530 nm). The level of ROS production corresponds to the average fluorescence intensity in the population of hemocytes, which is expressed in arbitrary units (a.u.).

The metabolic activity of hemocytes was evaluated using fluorescein diacetate (FDA, Invitrogen, ThermoFisher Scientific). This assay is based on the cleavage of the non-fluorescent FDA by intracellular enzymes (e.g., esterases) to yield fluorescent fluorescein. Briefly, 1 mL hemocyte samples was incubated with FDA at a final concentration of 12.5 µM for 15 min in the dark at room temperature. Fluorescence of the resulting product was then measured by flow cytometry using the FL1 channel (excitation 488 nm, emission 533/30 nm) [28].

### 2.6. Antioxidant Enzyme Activities and Oxidative Stress Markers

All chemicals were purchased from Sigma-Aldrich (USA). The tissue samples were thawed in an ice bath and homogenized in a cold 20 mM Tris–HCl buffer (pH 7.5) that included 0.5 mM EDTA. Supernatants were collected by centrifuging the homogenates for 20 min at 11,000× *g* at 4 °C [29] and were immediately used for enzyme activity determination.

For all enzymes, the activity was assessed in triplicate. Samples of gills were thawed in an ice bath and homogenized in a cold 20 mM Tris/HCl buffer (pH = 7.5) that included 0.5 mM ethylenediaminetetraacetic acid (EDTA). Supernatants were collected by centrifuging the homogenates at 20 min at 11,000× *g* at 4 °C and were immediately used for evaluation of enzyme activities. The activity of CAT was determined using the reaction between hydrogen peroxide and ammonium molybdate, which forms a yellow-colored complex at 405 nm [30]. The activity was expressed in μmol H_2_O_2_ min^−1^ mg protein^−1^. The method of Nishikimi et al. [31] based on the nitroblue tetrazolium assay was used to determine the activity of superoxide dismutase (SOD). Measurements were taken at 540 nm, and results were expressed in arbitrary units (a.u.). Protein content was analyzed using the Lowry method [32]. TBARS levels were calculated based on malondialdehyde (MDA) accumulation at 532 nm and were expressed as μmol MDA mg tissue-1, using the method described by Ohkawa et al. [33].

### 2.7. Immune and Antioxidant Gene Expression

In both experiments, hemocytes were collected from five mussels, with each mussel constituting an independent biological replicate (total n = 5). Total RNA was isolated from the hemocyte samples using the commercial RNA-Xtrac Plus kit (Bioinnlabs, Russia). The isolation procedure was performed strictly in accordance with the manufacturer’s protocol, which included a step of on-column DNase I treatment to eliminate genomic DNA contamination. The concentration and purity of the isolated total RNA were accurately determined using the Qubit 4 Fluorometer with the Qubit RNA HS Assay Kit (Thermo Fisher Scientific, Italy). To rigorously assess RNA integrity and the potential presence of degradation, all samples were subjected to analytical electrophoresis in a denaturing 1.5% agarose gel.

For reverse transcription, 500 ng of purified total RNA from each sample was used as a template for first-strand cDNA synthesis. The reaction was carried out using MMLV Reverse Transcriptase (Evrogen, Russia) according to the manufacturer’s protocol. The resulting cDNA samples were diluted with nuclease-free water and stored at −20 °C until further use in qPCR assays.

Expression profiles of selected transcripts were assessed by real-time qPCR using primer pairs reported in Table 1.

Quantitative real-time PCR analysis (RT-qPCR) was performed on a LightCycler^®^ 96 Instrument (Roche, Switzerland) using a qPCRmix-HS kit with SYBR Green I (Evrogen, Russia). The reaction mixture (total volume 15 μL) contained 1 μL of cDNA and 0.4 μM of each primer. The reaction conditions consisted of an initial denaturation at 95 °C for 3 min, followed by 40 cycles of 95 °C for 10 s, 60 °C for 10 s, and 72 °C for 15 s. Melting curve data were collected at 65–95 °C (0.5 °C/s). All reactions were performed with three technical replicates. For each run, a negative control without a template was included. Actin and *Elongation factor-1α* (*EF1*) were used as reference genes for real-time PCR analyses. The data analysis was carried out using Roche software v.1.1. Efficiencies of amplifications were determined by running a standard curve with serial dilutions of cDNA. For each measurement, a threshold cycle value (Cq) was determined as the fractional cycle number at which the fluorescence passes the fixed threshold. The descriptive statistics of the expression levels were computed for each candidate reference gene using the software package BestKeeper v.1 [37]. The relative expression levels of each target gene were normalized by calculating the geometric mean of the *ACT* and *EF1* genes using the ΔΔCt comparative method [38].

### 2.8. Statistical Analyses

The statistical analysis of data was conducted using R Studio version 4.4.1. The normality of the data was assessed through the Shapiro–Wilk test. The data were analyzed using a two-way ANOVA, followed by a Bonferroni multiple comparison post hoc test. The results were generated using the ggplot2 package in R (version 4.0.1).

## 3. Results and Discussion

**Figure 2:**
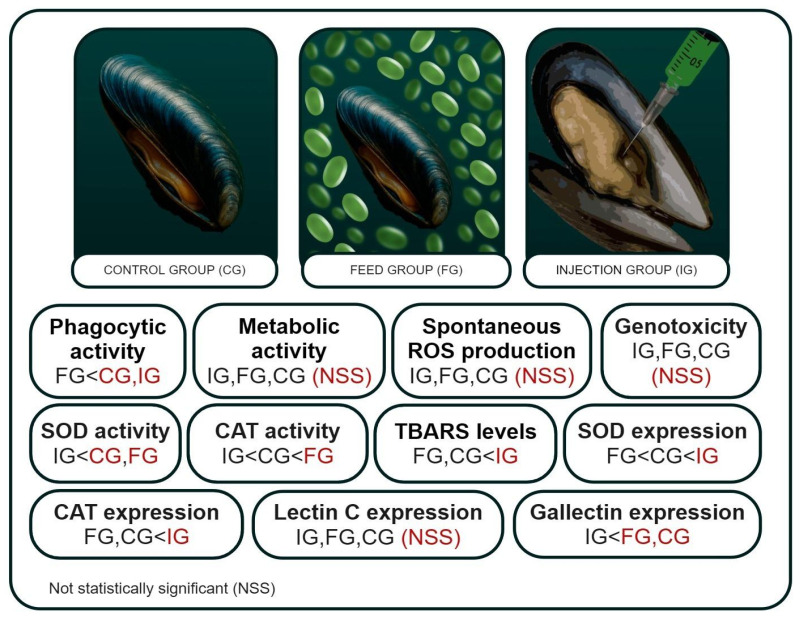
Immune parameters and indicators of antioxidant protection of the mussel *Mytilus galloprovincialis* when infected with microalgae *Coccomyxa* sp. in the control, feed, and injection groups.

Bivalve infection with various pathogens is a serious environmental and economic problem for aquaculture. In this work, the effect of the parasitic microalgae *Coccomyxa* sp. on the immune and antioxidant protection of the bivalve *M. galloprovincialis* was studied. The experiment involved three experimental groups: a control group injected with sterile seawater, an injection group administered a culture of *Coccomyxa* sp., and a feeding group exposed to a microalgae suspension. To understand the mechanisms of immune defense during invasion, the following immune parameters were studied: phagocytic and metabolic activity, intracellular production of ROS, and expression of *LEC* and *GAL* genes in mussel hemocytes (Figure 2). We also studied such indicators of antioxidant protection as DNA damage, gene expression and activity of SOD and CAT, and lipid peroxidation in gills and hemocytes.

### 3.1. Mortality Rate

**Figure 3:**
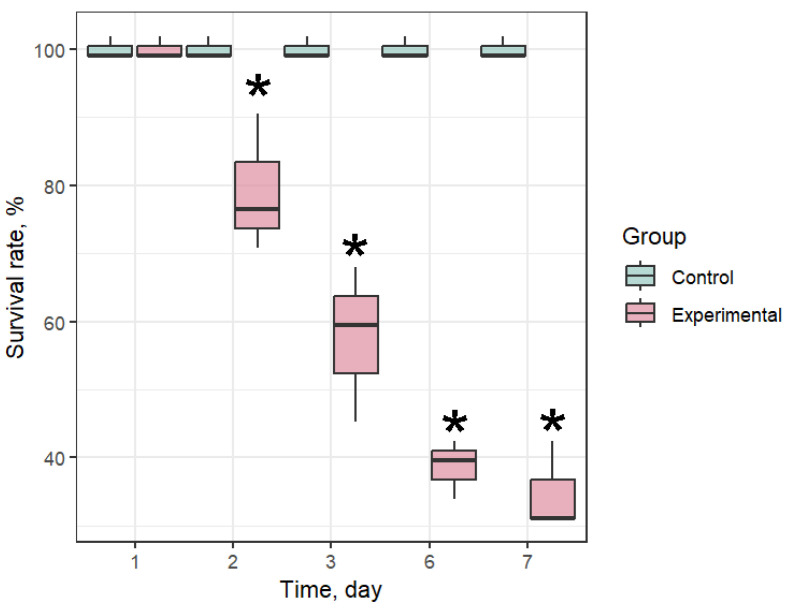
Survival rate of *Mytilus galloprovincialis* after infection with *Coccomyxa* sp. The differences were considered significant when *p* < 0.05 (*) based on Tukey’s HSD test.

Starting from the second day of the experiment, a high mortality rate in the infection groups was recorded (Figure 3). On the third day, the mortality rate reached 47%, and on the 7th day of the experiment it was 68%. At the same time, a 100% survival rate of mussels was recorded in the control group. The high mortality rate in the experimental groups highlights the potential destructiveness of *Coccomyxa* sp. for mariculture farms. Samples were taken from the surviving individuals of all groups on the 7th day of the experiment.

### 3.2. Infection

**Figure 4:**
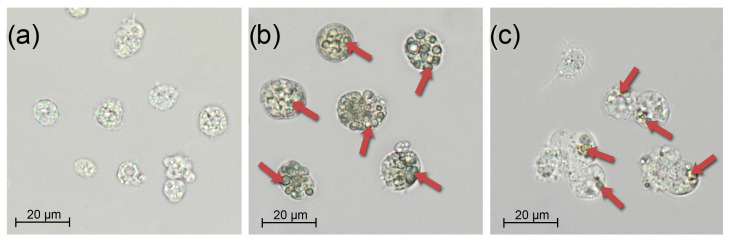
*Mytilus galloprovincialis* hemocytes under different challenge routes with *Coccomyxa* sp. on day 3: (**a**) Control, (**b**) Feed group, and (**c**) Injection group. Scale bar: 20 µm.

Independent of the infection type, undigested microalgae were detected in the bivalve hemocytes on the 7th day of the experiment. At the same time, in the feed group, the number of cells absorbed by one hemocyte exceeded 10, while in the injection group, their number was usually 2–3 cells per hemocyte (Figure 4). In the control group, the mussel hemocytes did not contain digested microalgae cells, and therefore, we can conclude that the hemolymph of the experimental groups contained parasitic microalgae.

### 3.3. Antioxidant Defense

**Figure 5:**
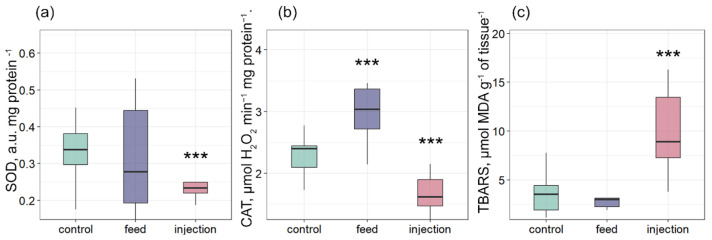
Antioxidant enzyme activities and oxidative stress markers in tissues of *Mytilus galloprovincialis* after infection with *Coccomyxa* sp.: (**a**) SOD activity, (**b**) CAT activity, and (**c**) TBARS levels. The differences were considered significant when *p* < 0.001 (***) based on Tukey’s HSD test.

It is known that mollusk infection with intracellular parasites can lead to an increase in ROS production [39,40]. Despite the importance of ROS production for mussel immune defense, excess can lead to oxidative stress [41]. SOD and CAT are the primary barriers to antioxidant protection [42]. The gills of bivalves perform many critically important functions for the organism, such as respiration and food filtration [43]. This functional role makes the gills a generally accepted target tissue for assessing the oxidative stress and oxidative damage level [44]. SOD activity was significantly lower in the injection group compared with the control, while no significant change was observed in the feed group (Figure 5a). CAT activity was significantly higher in the feed group and significantly lower in the injection group relative to the control (Figure 5b). TBARS levels were significantly elevated in the injection group compared with the control, whereas the feed group did not differ significantly from control values (Figure 5c). A similar result was shown in the work of Ter et al. [45], where infection of mollusk with *Vibrio mediterranei* by bacterial injection and addition to seawater also significantly increased the oxidation level. In the group with phagocytic infection, on the contrary, the activity of CAT was significantly increased, while the SOD activity and the TBARS level did not differ from the control. This indicated a compensatory activation of antioxidant protection.

**Figure 6:**
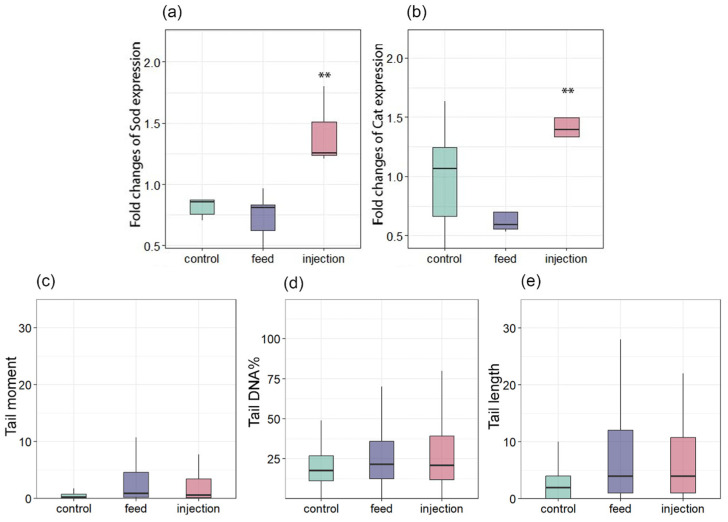
Changes in the expression of antioxidant genes in *Mytilus galloprovincialis* following infection with *Coccomyxa* sp.: (**a**) SOD and (**b**) CAT. DNA damage levels in hemocytes of *Mytilus galloprovincialis*, assessed by comet assay: (**c**) Tail moment, (**d**) Tail DNA%, and (**e**) Tail length. The relative expression levels of each target gene were normalized by calculating the geometric mean of the β-actin and ef1α genes using the ∆∆Ct comparative method. The differences were considered significant when *p* < 0.01 (**) based on Tukey’s HSD test.

In the present study, the level of CAT and SOD gene expression in hemocytes did not change significantly among the feeding group and control (Figure 6a,b). Conversely, the expression of SOD and CAT increased in the injection group. The comet assay was utilized to evaluate DNA damage in hemocytes. A consistent, though not statistically significant, increase in DNA damage was observed in both infected groups compared to the control. This trend was evident across all three comet assay parameters: mean tail moment, percentage of DNA in the tail (tail DNA%), and tail length were uniformly higher in the infected groups at all assessed time points (Figure 6c–e). While the differences did not reach statistical significance, likely due to biological variability, the directional consistency across these complementary metrics suggests a biologically relevant effect of infection on DNA integrity (Figure 6c–e). These results suggest a compensatory response in *M. galloprovincialis* within the range of physiological norms, without the involvement of additional mechanisms that regulate the antioxidant status through gene expression. Nevertheless, the increased expression of SOD and CAT indicates that transcription plays a role in regulating the antioxidant status of the injection group.

For example, *Meretrix meretrix* exhibited significant upregulation of *Cu/Zn*- and *Mn-SOD* within 24–48 h after injection with *Vibrio parahaemolyticus*, highlighting the importance of *SOD* isoforms in the initial immune response [46]. Similarly, bacterial injection in *Magallana gigas* (=*Crassostrea gigas*) rapidly induced *SOD* and *CAT* expression, peaking at 3–12 h, which supports their use as early biomarkers of immune activation [47]. A comparable scenario was reported in *Ruditapes philippinarum* infected with *V. tapetis*: early-stage infection induced subtle changes in antioxidant enzyme activity. However, resistant individuals later mounted a robust ROS-scavenging response, preventing clinical disease [48]. Thus, the stability of *SOD* and *CAT* expression in the feeding group may reflect successful mitigation of oxidative insult at sub-threshold levels. However, hemocytes, though central to systemic immunity, may not fully represent oxidative stress in primary barrier tissues, such as gills, which are highly susceptible to environmental pathogens and pollutants [48]. Nevertheless, the rapid transcriptional upregulation of *SOD* and *CAT* in the injection group likely prepares hemocytes for the quick deployment of enzymes should oxidative stress escalate. This strategy has been observed in multiple mussel species under acute stressors [49].

### 3.4. Immune Response

**Figure 7:**
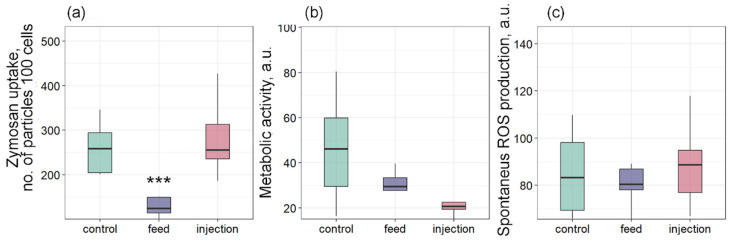
Effects of *Coccomyxa* sp. infection on phagocytic activity, metabolic activity, and reactive oxygen species (ROS) production in hemocytes of *Mytilus galloprovincialis*: (**a**) Phagocytic activity, (**b**) metabolic activity, and (**c**) spontaneous ROS production. The differences were considered significant when *p* < 0.001 (***) based on Tukey’s HSD test.

Bivalves have a limited set of mechanisms to control and reduce the negative effects of pathogens. Phagocytosis is one such process that allows cells to absorb and destroy foreign particles that threaten the organism’s integrity [50,51]. The penetration of exogenous pathogens causes hemocyte migration and phagocytic uptake of the pathogen [52]. The absorbed material is enzymatically degraded by the endosome and lysosome system [52]. In our study, phagocytic activity was significantly lower in the feed group compared to the control (Figure 7a). The phagocytosis activity may depend on the availability of food. For example, in the work by Le Guernic et al. [53], the bivalve phagocytic activity was significantly lower in the group receiving 100% nutrition compared to the group completely deprived of food. The authors attribute such changes in the observed parameter to the ability of mussel hemocytes to regulate the number of phagocytic particles depending on food availability. A similar result was obtained in an in vitro study by Bogacheva et al. [19], where phagocytic activity was significantly reduced in *M. galloprovincialis* hemocytes incubated with *Coccomyxa* sp. cells. This largely corresponds to the Stevenson & South [23] study, in which the authors found hemocytes with phagocytosed, undigested *C. parasitica* cells. A similar effect was also observed when toxic algae *Prorocentrum minimum* were exposed to bivalves, in which phagocytosis suppression was accompanied by massive infiltration of hemocytes into the intestine [54]. Interestingly, when microalgae were injected, phagocytosis was not suppressed, as when administered by filtration with food. This may indicate that mussel hemocytes are able to recognize microalgae cells as foreign when directly injected into a muscle, but the effectiveness of this recognition decreases with natural infection pathways.

Metabolic activity showed a decreasing trend in both infected groups but did not differ significantly from the control (Figure 7b). Similarly, spontaneous ROS production did not differ significantly among the three groups (Figure 7c). ROS molecules are involved in many biochemical reactions of the body and are also used as a secondary marker of immune system activity [50]. For example, a significant increase in ROS production was observed in mollusks infected with *Vibrio* sp. [39,40,55]. In a study by Auguste et al. [56], both in vivo and in vitro infection of *M. galloprovincialis* mussels with two strains of *Malaciobacter marinus* caused a significant increase in ROS production. This result also indicates an inhibition of the immune response in *M. galloprovincialis* after infection with the parasitic microalgae *Coccomyxa* sp. It is important to note that in the first hours of infection, an increase in ROS production is observed in mussel hemocytes, as shown in our work [19]. This acute oxidative response is typical of the initial phase of pathogen recognition and indicates the readiness of the mussel immune system for a rapid reaction [57,58]. However, in the present in vivo study, where the assessment was performed on the 7th day after the introduction of the parasitic microalgae, spontaneous ROS production and metabolic activity did not differ from the control in any of the groups. This suggests that by this time, a chronic phase of infection is developing, during which the immune system is suppressed by the parasite. Thus, *Coccomyxa* sp. appears to be able not only to initiate an acute immune response, but also to effectively suppress it in the long term.

**Figure 8:**
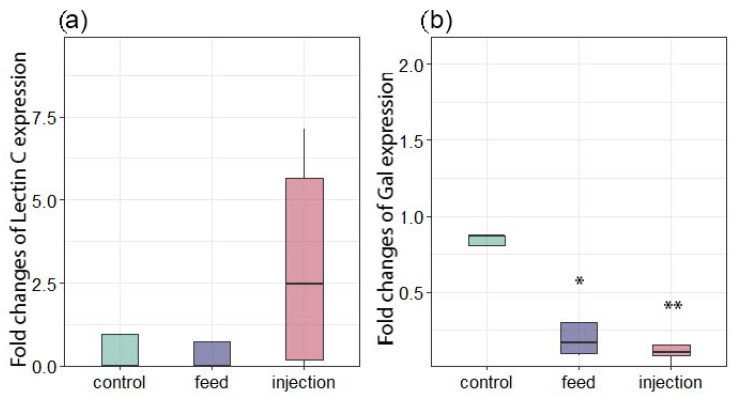
Changes in the expression of antioxidant and immune-related genes in *Mytilus galloprovincialis* following infection with *Coccomyxa* sp. (**a**) Lectin C. (**b**) Gal. The relative expression levels of each target gene were normalized by calculating the geometric mean of the β-actin and ef1α genes using the ∆∆Ct comparative method. The differences were considered significant when *p* < 0.05 (*) and *p* < 0.01 (**) based on Tukey’s HSD test.

In addition to the cellular immune response, the innate immune system relies on a relatively small number of receptors that can recognize broad molecular patterns associated with pathogens (PAMPs) and activate both cellular and humoral responses. Lectins are considered an important pattern recognition receptor (PRR) [59]. Lectins are also able to differentially bind certain microalgae types in the mollusks’ food organs, participating in the selection of food particles [60]. Expression of Lectin C did not differ significantly among the three groups (Figure 8a). In contrast, Gal expression was significantly downregulated in the injection group relative to the control, while the feed group did not differ significantly from the control (Figure 8b). A similar result was observed in the work by Yamaura et al. [61], in which the authors attributed this to a decrease in the adhesive ability of cells in acute bacterial infection. In a study by Yang et al. [61], the expression of galectin CgGal-CUB increased significantly after infection of *M. gigas* with the pathogen *V. splendidus*, which was also accompanied by an increase in phagocytic activity. Also, injection of *Vibrio alginolyticus* caused an increase in galectin expression in the mussel *Mytilus coruscus* [42]. Since in our study we observed a decrease in the galectin expression, a gene associated with phagocytosis, apoptosis, and inflammation modulation, in both groups of mussel interactions with the parasitic microalgae *Coccomyxa* sp., this may indicate the development of immunosuppression in *M. galloprovincialis*. Such galectin suppression may be a key immunosuppression mechanism used by the parasite to evade the host’s immune response.

## 4. Conclusions

This study is the first to demonstrate under controlled experimental conditions that the green parasitic microalga *Coccomyxa* sp., originally isolated from the Kuril horse mussel in the Sea of Japan, can effectively infect the Mediterranean mussel *M. galloprovincialis* at a salinity of 18‰, which is typical of the Black Sea. Regardless of the infection route—through dietary intake (filtration of algae-containing feed) or direct microinjection into the adductor muscle—mortality reached 68% by day 7 in both experimental groups, while all individuals in the control group remained viable. The mode of infection significantly influenced the host’s immune and antioxidant responses. In the feeding group, the phagocytic activity of hemocytes was markedly suppressed, likely due to the parasite’s ability to mimic food particles and evade immune recognition. In contrast, injection did not suppress phagocytosis. However, it led to significant downregulation of the galectin gene, which is a key pattern recognition receptor involved in pathogen recognition, phagocytosis, and modulation of inflammatory responses.

Although the expression of genes encoding the antioxidant enzymes SOD and CAT increased in hemocytes following injection, the enzymatic activity of both SOD and CAT decreased significantly in gill tissues. This was accompanied by elevated levels of lipid peroxidation, which is indicative of localized oxidative stress. Conversely, catalase activity in the gills of the feeding group was significantly increased, suggesting a compensatory antioxidant response. DNA damage in hemocytes showed an upward trend in both infected groups compared to the control group across all comet assay parameters (tail moment, percent tail DNA, and tail length), though these differences were not statistically significant. This implies that, at the time of sampling (day 7), cellular integrity remained within physiological limits and that no severe genotoxic stress had occurred.

Taken together, these findings confirm that *Coccomyxa* sp. is a highly virulent and adaptable parasite that can infect *M. galloprovincialis* under low-salinity conditions. It can also actively modulate the host’s immune and antioxidant defense systems. The high mortality rate and disruption of key physiological pathways highlight the serious threat that this microalga poses to mussel aquaculture in the Black Sea and other brackish water regions, particularly in the context of climate change, species invasions, and the expansion of non-native pathogens into new ecosystems. Future studies could focus on a more in-depth analysis of oxidative stress, including the spatiotemporal dynamics of its markers across different tissues during *Coccomyxa* sp. infection, as well as quantitative and qualitative assessment of oxidative damage, such as lipid peroxidation.

## Figures and Tables

**Table 1:** Primer sequence of target and reference genes for RT-qPCR.

Est Name	Forward 5′–3′	Reverse 5′–3′	Gene Accession Numbers	Source
Actin	CTCTTGATTTCGAGCAGGAAA	AGGATGGTTGGAATAATGATT	AF157491	[34]
Elongation factor-1α (EF1)	GTGGGATGATGTTGATATAG	CCATTGCCTATAGCATTTAC	AB162021	[35]
Superoxide dismutase	AACAGTCGCTTTCAGTCAAC	TACATTTCCCAGATCACCAAC	FM177867	[36]
Catalase	TGCTCTGGGATTTCATTAC	CAGCACTCAGACATTTTATAC	AY743716	[36]
Galectin8	GCGTTGCTCCAGCCCATTCAGA	AGCCCAGGCATTGTTTTTCAGCG	UYJE01007246	designed in this study
Lectin c-type	GCCGTGGAGCCAGTATTTCTTTCCG	GGGGGAGACAGTGTTGCGTGTT	UYJE01002995	designed in this study

## Data Availability

The datasets generated and/or analyzed during the current study are available from the corresponding author on request.

## Abbreviations

The following abbreviations are used in this manuscript:

A.U.	Arbitrary units
CAT	Catalase
Cq	Threshold cycle value
DCFH-DA	2′,7′-dichlorodihydrofluorescein diacetate
DNA	Deoxyribonucleic acid
EF1	Elongation factor-1α
FDA	Fluorescein Diacetate
GAL	Galectin
LEC	Lectin
PCR	Polymerase chain reaction
PI	Propidium iodide
ROS	Reactive oxygen species
RT-qPCR	Quantitative real-time PCR
SOD	Superoxide dismutase
TBARS	Thiobarbituric acid reactive substances

## References

[1] Food and Agriculture Organization (2020). The State of World Fisheries and Aquaculture 2020: Sustainability in Action.

[2] Tan K., Xu P., Huang L., Luo C., Huang J., Fazhan H., Kwan K.Y. (2024). Effects of bivalve aquaculture on plankton and benthic community. Sci. Total Environ..

[3] Lins D.M., Zbawicka M., Wenne R., Poćwierz-Kotus A., Molina J.R., Alves L.P., Rocha R.M. (2021). Ecology and genetics of *Mytilus galloprovincialis*: A threat to bivalve aquaculture in southern Brazil. Aquaculture.

[4] Peeler E.J., Oidtmann B.C., Midtlyng P.J., Miossec L., Gozlan R.E. (2011). Non-native aquatic animals introductions have driven disease emergence in Europe. Biol. Invasions.

[5] Beaury E.M., Fusco E.J., Jackson M.R., Laginhas B.B., Morelli T.L., Allen J.M., Pasquarella V.J., Bradley B.A. (2020). Incorporating climate change into invasive species management: Insights from managers. Biol. Invasions.

[6] Costello K.E., Lynch S.A., O’Riordan R.M., McAllen R., Culloty S.C. (2021). The Importance of Marine Bivalves in Invasive Host–Parasite Introductions. Front. Mar. Sci..

[7] Alfjorden A., Onut-Brännström I., Wengström N., Kristmundsson A., Jamy M., Persson B.D., Burki F. (2024). Identification of a new gregarine parasite associated with mass mortality events of freshwater pearl mussels (*Margaritifera margaritifera*) in Sweden. J. Eukaryot. Microbiol..

[8] Sanil N.K., Suja G., Asokan P.K. (2025). Diseases of Bivalve Molluscs. Aquatic Animal Health Management.

[9] Du X., Sun J., Ju H., Xu Z., Tang X., Fang X., Chang M.S. (2025). Metazoan parasites associated with marine mollusks inhabiting the China Seas: A review. Front. Mar. Sci..

[10] Sadikaj R., Arapi D., Caka G., Ylli A., Puto K., Ngresi A., Meli E. (2025). An assessment about some parasitic species in a mediterranean mussel (*Mytilus galloprovincialis* L., 1819) population from the north-eastern coast of Karaburun Peninsula (south-eastren coast of Adriatic Sea). Int. J. Ecosyst. Ecol. Sci..

[11] Lattos A., Papadopoulos D.K., Feidantsis K., Giantsis I.A., Georgoulis I., Karagiannis D., Michaelidis B. (2023). Antioxidant defense of *Mytilus galloprovincialis* mussels induced by marine heatwaves in correlation with *Marteilia* pathogen presence. Fishes.

[12] Azizan A., Venter L., Alfaro A.C. (2024). Biomarkers of mussel exposure to *Vibrionaceae*: A review. Aquac. Int..

[13] Kim S.H., Kim H.J., Bathige S.D.N., Kim S., Park K.I. (2025). Strain-specific virulence of *Perkinsus marinus* and related species in Eastern oysters: A comprehensive analysis of immune responses and mortality. Fish Shellfish Immunol..

[14] Ma W., Zeng W., Zhang D., Zhou Y., Huang Y., Hong Y. (2025). Oxidative Stress in Aquaculture: Pathogenic Mechanisms and Preventive Strategies in Farmed Aquatic Animals. Curr. Issues Mol. Biol..

[15] Belzile C., Gosselin M. (2015). Free-living stage of the unicellular algae *Coccomyxa* sp. parasite of the blue mussel (*Mytilus edulis*): Low-light adaptation, capacity for growth at a very wide salinity range and tolerance to low pH. J. Invertebr. Pathol..

[16] Zuykov M., Anderson J., Archambault P., Dufresne F., Pelletier E. (2018). *Mytilus trossulus* and hybrid (*M. edulis*-*M. trossulus*)—New hosts organisms for pathogenic microalgae *Coccomyxa* sp. from the Estuary and northwestern Gulf of St. Lawrence, Canada. J. Invertebr. Pathol..

[17] Sokolnikova Y., Magarlamov T., Stenkova A., Kumeiko V. (2016). Permanent culture and parasitic impact of the microalga Coccomyxa parasitica, isolated from horse mussel Modiolus kurilensis. J. Invertebr. Pathol..

[18] Tumas A.V., Slatvinskaya V.A., Kumeiko V.V., Sokolnikova Y.N. (2024). Study of the Impact of the Parasitic Microalgae *Coccomyxa parasitica* on the Health of Bivalve *Modiolus kurilensis*. Microorganisms.

[19] Bogacheva E.A., Kukhareva T.A., Tkachuk A.A., Kladchenko E.S., Lavrichenko D.S., Podolskaya M.S., Andreyeva A.Y., Chelebieva E.S. (2024). Reactions of the Hemocytes Cellular Immunity of the Mediterranean Mussel *Mytilus galloprovincialis* to Invasion by the Green Microalga *Coccomyxa parasitica* (In Vitro). Oceanology.

[20] Zuykov M., Kolyuchkina G., Archambault P., Gosselin M., Anderson J., McKindsey C.W., Spiers G., Schindler M. (2020). Shell deformity as a marker for retrospective detection of a pathogenic unicellular alga, *Coccomyxa* sp., in mytilid mussels: A first case study and research agenda. J. Invertebr. Pathol..

[21] Zuykov M., Belzile C., Lemaire N., Gosselin M., Dufresne F., Pelletier E. (2014). First record of the green microalgae Coccomyxa sp. in blue mussel *Mytilus edulis* (L.) from the Lower St. Lawrence Estuary (Québec, Canada). J. Invertebr. Pathol..

[22] Sokolnikova Y., Tumas A., Stenkova A., Slatvinskaya V., Magarlamov T., Smagina E. (2022). Novel species of parasitic green microalgae *Coccomyxa veronica* sp. nov. infects *Anadara broughtonii* from the Sea of Japan. Symbiosis.

[23] Stevenson R.N., South G.R. (1974). *Coccomyxa parasitica* sp. nov. (Coccomyxaceae, Chlorococcales), a parasite of giant scallops in Newfoundland. Br. Phycol. J..

[24] Kladchenko E.S., Lavrichenko D.S., Bogacheva E.A., Chelebieva E.S. (2025). Effects of hyposalinity stress on the physiological state of the marine microalgae *Coccomyxa parasitica*. Reg. Stud. Mar. Sci..

[25] Guillard R.R., Ryther J.H. (1962). Studies of marine planktonic diatoms: I. Cyclotella nana Hustedt, and Detonula confervacea (Cleve) Gran. Can. J. Microbiol..

[26] Kan Y., Pan J. (2010). A one-shot solution to bacterial and fungal contamination in the green alga *Chlamydomonas reinhardtii* culture by using an antibiotic cocktail 1. J. Phycol..

[27] Møller P., Azqueta A., Boutet-Robinet E., Koppen G., Bonassi S., Milić M., Gajski G., Costa S., Teixeira J.P., Pereira C.C. (2020). Minimum Information for Reporting on the Comet Assay (MIRCA): Recommendations for describing comet assay procedures and results. Nat. Protoc..

[28] Michelangeli M.E., Brandsma S.H., Margalef M., Forsman E., Kuehr S., Spanu D., Gomes T. (2025). Chemical leachates from car tyre granulates and PET bottles induce toxic effects on *Mytilus edulis* haemocytes. Environ. Chem. Ecotoxicol..

[29] Cossi P.F., Herbert L.T., Yusseppone M.S., Pérez A.F., Kristoff G. (2020). Toxicity evaluation of the active ingredient acetamiprid and a commercial formulation (Assail^®^ 70) on the non-target gastropod *Biomphalaria straminea* (Mollusca: Planorbidae). Ecotoxicol. Environ. Saf..

[30] Goth L. (1991). A simple method for determination of serum catalase activity and revision of reference range. Clin. Chim. Acta.

[31] Nishikimi M., Rao N.A., Yagi K. (1972). The occurrence of superoxide anion in the reaction of reduced phenazine methosulfate and molecular oxygen. Biochem. Biophys. Res. Commun..

[32] Lowry O.H., Rosebrough N.J., Farr A.L., Randall R.J. (1951). Protein measurement with the Folin phenol reagent. J. Biol. Chem..

[33] Ohkawa H., Ohishi N., Yagi K. (1979). Assay for lipid peroxides in animal tissues by thiobarbituric acid reaction. Anal. Biochem..

[34] Dondero F., Piacentini L., Marsano F., Rebelo M., Vergani L., Venier P., Viarengo A. (2006). Gene transcription profiling in pollutant exposed mussels (*Mytilus* spp.) using a new low-density oligonucleotide microarray. Gene.

[35] Woo S., Jeon H.Y., Kim S.R., Yum S. (2011). Differentially displayed genes with oxygen depletion stress and transcriptional responses in the marine mussel, *Mytilus galloprovincialis*. Comp. Biochem. Physiol. Part D Genom. Proteom..

[36] Woo S., Denis V., Won H., Shin K., Lee G., Lee T.K., Yum S. (2013). Expressions of oxidative stress-related genes and antioxidant enzyme activities in *Mytilus galloprovincialis* (Bivalvia, Mollusca) exposed to hypoxia. Zool. Stud..

[37] Pfaffl M.W., Tichopad A., Prgomet C., Neuvians T.P. (2004). Determination of stable housekeeping genes, differentially regulated target genes and sample integrity: BestKeeper–Excel-based tool using pair-wise correlations. Biotechnol. Lett..

[38] Livak K.J., Schmittgen T.D. (2001). Analysis of relative gene expression data using real-time quantitative PCR and the the 2^−ΔΔCT^ Method. Methods.

[39] Nguyen T.V., Alfaro A.C., Young T., Ravi S., Merien F. (2018). Metabolomics study of immune responses of New Zealand greenshell™ mussels (*Perna canaliculus*) infected with pathogenic *Vibrio* sp.. Mar. Biotechnol..

[40] Balbi T., Auguste M., Cortese K., Montagna M., Borello A., Pruzzo C., Vezzulli L., Canesi L. (2019). Responses of *Mytilus galloprovincialis* to challenge with the emerging marine pathogen *Vibrio coralliilyticus*. Fish Shellfish Immunol..

[41] Kladchenko E.S., Tkachuk A.A., Podolskaya M.S., Andreyeva A.Y. (2024). ROS production and mitochondrial membrane potential in hemocytes of marine bivalves, *Mytilus galloprovincialis* and *Magallana gigas*, under hypoosmotic stress. Comp. Biochem. Physiol. Part B Biochem. Mol. Biol..

[42] Li R., Zhao R., Li S., Yang Y., Li L., Wu K., Di Y. (2025). Systemic protection through enhanced immunity and antioxidant defenses in immune-primed *Mytilus coruscus*: Insights from cell/tissue-specific analyses. Fish Shellfish Immunol..

[43] Bouallegui Y., Ben Younes R., Bellamine H., Oueslati R. (2017). Histopathology and analyses of inflammation intensity in the gills of mussels exposed to silver nanoparticles: Role of nanoparticle size, exposure time, and uptake pathways. Toxicol. Mech. Methods.

[44] Wang X., Shao S., Zhang T., Zhang Q., Yang D., Zhao J. (2023). Effects of exposure to nanoplastics on the gill of mussels *Mytilus galloprovincialis*: An integrated perspective from multiple biomarkers. Mar. Environ. Res..

[45] Ter Ü., Gürkan S.E., Gürkan M., Kunili I.E., Aksoy E. (2024). Pathological and oxidative stress responses of *Mytilus galloprovincialis* to *Vibrio mediterranei* infection: An in vivo challenge. Fish Shellfish Immunol..

[46] Lu X., Wang C., Liu B. (2015). The role of Cu/Zn-SOD and Mn-SOD in the immune response to oxidative stress and pathogen challenge in the clam *Meretrix meretrix*. Fish Shellfish Immunol..

[47] Meng J., Zhang L., Huang B., Li L., Zhang G. (2015). Comparative analysis of oyster (*Crassostrea gigas*) immune responses under challenge by different *Vibrio* strains and conditions. Molluscan Res..

[48] Richard G., Guérard F., Corporeau C., Lambert C., Paillard C., Pernet F. (2016). Metabolic responses of clam *Ruditapes philippinarum* exposed to its pathogen *Vibrio tapetis* in relation to diet. Dev. Comp. Immunol..

[49] Wu J., Bao M., Ge D., Huo L., Lv Z., Chi C., Liao Z., Liu H. (2017). The expression of superoxide dismutase in *Mytilus coruscus* under various stressors. Fish Shellfish Immunol..

[50] De la Ballina N.R., Maresca F., Cao A., Villalba A. (2022). Bivalve haemocyte subpopulations: A review. Front. Immunol..

[51] Matozzo V., Brunelli N., Cima F. (2025). The underrated immune role of bivalve ‘serous cells’: New insight from inflammatory responses of the Manila clam Ruditapes philippinarum. Fish Shellfish Immunol..

[52] Hartenstein V., Martinez P. (2019). Phagocytosis in cellular defense and nutrition: A food-centered approach to the evolution of macrophages. Cell Tissue Res..

[53] Le Guernic A., Felix C., Bigot A., David E., Dedourge-Geffard O., Geffard A., Betoulle S. (2015). Food deprivation and modulation of hemocyte activity in the zebra mussel (*Dreissena polymorpha*). J. Shellfish. Res..

[54] Hégaret H., da Silva P.M., Wikfors G.H., Haberkorn H., Shumway S.E., Soudant P. (2011). In vitro interactions between several species of harmful algae and haemocytes of bivalve molluscs. Cell Biol. Toxicol..

[55] Canesi L., Barmo C., Fabbri R., Ciacci C., Vergani L., Roch P., Gallo G. (2010). Effects of *Vibrio* challenge on digestive gland biomarkers and antioxidant gene expression in *Mytilus galloprovincialis*. Comp. Biochem. Physiol. Part C Toxicol. Pharmacol..

[56] Auguste M., Rahman F.U., Balbi T., Leonessi M., Oliveri C., Bellese G., Vezzulli L., Furones D., Canesi L. (2022). Responses of *Mytilus galloprovincialis* to challenge with environmental isolates of the potential emerging pathogen *Malaciobacter marinus*. Fish Shellfish Immunol..

[57] Ericson J.A., Venter L., Welford M.R., Kumanan K., Alfaro A.C., Ragg N.L. (2022). Effects of seawater temperature and acute *Vibrio* sp. challenge on the haemolymph immune and metabolic responses of adult mussels (*Perna canaliculus*). Fish Shellfish Immunol..

[58] Kang Y.S., Kim Y.M., Park K.I., Cho S.K., Choi K.S., Cho M. (2006). Analysis of EST and lectin expressions in hemocytes of Manila clams (*Ruditapes philippinarum*) (Bivalvia: Mollusca) infected with *Perkinsus olseni*. Dev. Comp. Immunol..

[59] Allam S., Allam B., Pales Espinosa E. (2021). Regulation of mucosal lectins in the oyster *Crassostrea virginica* in response to food availability and environmental factors. J. Molluscan Stud..

[60] Yamaura K., Takahashi K.G., Suzuki T. (2008). Identification and tissue expression analysis of C-type lectin and galectin in the Pacific oyster, *Crassostrea gigas*. Comp. Biochem. Physiol. Part B Biochem. Mol. Biol..

[61] Yang W., Sun J., Leng J., Li Y., Guo Q., Wang L., Song L. (2024). A novel lectin with a distinct Gal_Lectin and CUB domain mediates haemocyte phagocytosis in oyster *Crassostrea gigas*. Dev. Comp. Immunol..

